# The Effectiveness and Mechanisms of Action of App-Based Interventions for Improving Mental Health and Workplace Well-Being: Randomized Controlled Trial

**DOI:** 10.2196/91564

**Published:** 2026-04-27

**Authors:** Alexander MacLellan, Graeme Fairchild, Katherine S Button

**Affiliations:** 1 Department of Psychology, University of Bath, Claverton Down, Bath, England, BA2 7AY, United Kingdom, 44 01225 388388

**Keywords:** cognitive control, depression, anxiety, computerized cognitive training, digital mental health

## Abstract

**Background:**

Depression is the most common mental health disorder worldwide and frequently leads to workplace absence. As face-to-face treatment can be difficult to access, app-based interventions are a popular solution, although their effectiveness in working populations and their mechanisms of action are unclear. Deficits in executive function may contribute to the onset and maintenance of depression, and executive function training is proposed to improve symptoms by enhancing executive function. Responders to cognitive behavioral therapy (CBT) show improvements in executive function, suggesting that this may be one mechanism of action.

**Objective:**

This study investigated the effectiveness of app-based interventions (executive function or CBT-based) for reducing depressive and anxiety symptoms and improving workplace well-being, and assessed whether changes in executive function mediated improvements.

**Methods:**

A total of 228 participants (147 female participants) with mild-to-moderate symptoms of depression and anxiety were recruited online and randomly assigned to a waitlist control group, an executive function training group (NeuroNation app, Synaptikon GmbH), or a self-guided CBT group (Moodfit app, Roble Ridge LLC) for a 4-week intervention period. Participants assigned to the active intervention groups were asked to use their apps a minimum of 21 times during the intervention. Participants completed measures of depressive symptoms, anxiety symptoms, and workplace well-being, and a working memory task at baseline, postintervention, and follow-up (12 weeks).

**Results:**

Executive function training reduced anxiety (β=−2.79; *P*=.004) and depressive (β=−2.77; *P*=.02) symptoms at follow-up but not at postintervention, and it did not affect workplace well-being. There were no reductions in depressive or anxiety symptoms in the self-guided CBT group, though workplace well-being was improved at postintervention (β=3.72; *P*=.02) and follow-up (β=4.46; *P*=.02). Improvements in executive function did not mediate intervention-related changes in symptoms or workplace well-being. Self-reported adherence rates were high (executive function training: 48/54, 89%; self-guided CBT: 52/54, 96%), although attrition was high at follow-up (58% missing).

**Conclusions:**

These results suggest that app-based executive function training may be effective at managing symptoms of anxiety and depression in a working population, while self-guided CBT apps may improve workplace well-being. However, improving executive function did not appear to be a mechanism of action of either intervention.

## Introduction

Depression is the most common mental health disorder worldwide [1] and is now the most common factor leading to lower workplace productivity [2] and absence from work [3]. Targeting people with subclinical depression (ie, those “at risk”) is considered a priority for preventing future episodes [4], and as subclinical depression and anxiety may impact workplace performance, companies are increasingly turning to smartphone apps targeting mental health and well-being to support their employees [5]. These typically draw on principles from cognitive behavioral therapy (CBT) or other psychological therapies, and purport to target the core characteristics of depression, such as low mood, negative biases, and anhedonia. Though not directly targeted by these apps, absenteeism from the workplace, low productivity, and engagement in unhealthy working patterns are linked to deficits in executive function in depressed populations, suggesting that cognitive function must also be targeted to improve work-related outcomes [6].

Cognitive models suggest that impaired executive functions, such as inhibitory control, attention, and working memory, underlie both the onset and maintenance of depression [7] and anxiety [8]. These models suggest that impairments in executive function result in a reduced ability to inhibit negative thoughts, shift attention away from them, or update negative information currently held in working memory in response to new, potentially positive, information, leading to the onset or maintenance of symptoms [9]. In the working population, executive function is positively related to employment status and the ability to effectively balance tasks and responsibilities [10]. Deficits in executive function have been identified in populations with depression [11] and anxiety [8], with the severity of symptoms being correlated with the magnitude of impairment [12]. Given that executive function has been found to have a reciprocal relationship with depressive symptoms [13], with impairments in executive function predicting future depressive symptoms [13,14], continuing into remission, and worsening with repeated episodes [15], executive function is a key target for intervention.

Computerized interventions targeting different facets of executive function, such as cognitive control, aim to target putative neurocognitive mechanisms underlying mental health disorders. By enhancing executive function, it is hypothesized that the affected individual is better able to inhibit or shift attention away from negative information, reducing the cognitive symptoms of depression and anxiety and improving mood. The appeal of executive function training lies in the promise of a low-cost, easily accessible intervention that translates findings from basic research to clinical utility. Accordingly, systematic reviews have found broadly positive effects of computerized executive function training on depressive symptoms [7,16]. For example, a commercially available app, NeuroNation (Synaptikon GmbH), was found to improve working memory capacity after 21 sessions of training [17]. However, the malleability of executive function has been debated [18], and although research has found that some aspects of executive function can be improved by training (eg, working memory) [16], this has not been consistently shown (eg, working memory did not improve after 2 weeks of training [19]). There are also concerns regarding whether the effects of these interventions generalize beyond the trained tasks [18]. In addition, owing to a lack of follow-up data, the duration of effects is unknown [16]. Overall, the literature has yielded mixed findings regarding the effects of executive function training, and explicit tests of the mechanisms of action would help in resolving this debate [18].

Self-paced digital CBT offers another low-cost, accessible intervention, and it has been proposed as a solution to situations where people are currently on waiting lists for treatment or where clinicians are engaged in “watchful waiting” [20]. Executive function has been found to predict the response to CBT [21,22], enhance compliance with CBT homework practices [23], and improve after successful treatment [24], which may be due to CBT targeting and restructuring negative thoughts, thereby reducing the burden on executive function, which can support further change. Therefore, improving executive function may be a common pathway by which psychological interventions reduce the symptoms of depression and anxiety. Systematic reviews have found reductions in depressive symptoms with CBT delivered via automated text messages [25] or via CBT-based apps [26], though there is a lack of research investigating their effects on executive function. As with studies on executive function training, there are also concerns regarding the effectiveness of these interventions, given notably high attrition rates [27] and a lack of research in real-world settings, with studies often conducted in undergraduate populations or often excluding participants who do not engage with the intervention. It is not clear whether these results would generalize to working adults who face, for example, multiple time pressures.

Positive outcomes, such as well-being, are equally important outcomes of interest, both as predictors of future depressive episodes [28] and as intervention targets [29]. Work-related stress and well-being also predict future episodes of depression [30], though they are rarely included as outcomes in intervention research. Understanding how interventions affect not only negative outcomes but also positive outcomes could provide a more complete picture of an intervention’s effectiveness and clarify for whom it is most effective. Additionally, though anxiety is highly prevalent in the working population and comorbid with depression, it is relatively underresearched compared with depression [31]. As anxiety is also associated with long-term absenteeism and reduced productivity [32], exploring the effects of app-based interventions on anxiety symptoms and the potential mechanisms of action is of interest. Specifically, drawing on the Research Domain Criteria framework, executive function may be a transdiagnostic mechanism of action [33] through which diverse interventions improve mental health and daily functioning. For example, improving executive function may result in more efficient filtering and regulation of negative information in working memory, promoting more neutral or positive interpretations and improving mood.

We therefore aimed to test the effectiveness of an executive function training app and a CBT-based app on mental health, well-being, and executive functioning in a working adult sample at risk of depression and anxiety disorders. We also aimed to test whether changes in these outcomes are mediated by changes in executive function, which would support models proposing that executive function is a common pathway through which treatments may work. This study, therefore, is a 3-arm randomized controlled trial investigating the effectiveness of an executive function training app (NeuroNation) and a CBT-based app (Moodfit, Roble Ridge LLC), compared with a waitlist control. NeuroNation delivers a variety of gamified executive function tasks each day, with gamification proposed to improve intrinsic motivation and training protocol adherence [34]. Moodfit is a self-paced mental health app using a variety of techniques from CBT, such as cognitive restructuring, as well as mindfulness and positive psychology. By assessing both negative symptoms, such as mood and anxiety, and positive outcomes, such as workplace well-being, at baseline, postintervention (after using the apps for 4 weeks), and follow-up (12 weeks), we aimed to provide a more comprehensive assessment of their effectiveness in real-world settings. We therefore tested the following hypotheses:

- Hypothesis 1: There will be greater reductions in depressive (hypothesis 1a) and anxiety (hypothesis 1b) symptoms and greater increases in workplace well-being (hypothesis 1c) in the executive function training and self-guided CBT groups at postintervention and follow-up (12 weeks), compared with the findings in the waitlist control group.
- Hypothesis 2: Intervention-related changes in depressive (hypothesis 2a) and anxiety (hypothesis 2b) symptoms and workplace well-being (hypothesis 2c) will be mediated by improvements in executive function.

## Methods

### Transparency and Openness

Materials, code, and preregistration data for this study are available on the Open Science Framework (OSF) [35]. This study was preregistered on the OSF prior to commencing data collection, and data are available via the University of Bath’s Research Data Archive [36]. The preregistered sample size rationale is provided in Multimedia Appendix 1.

### Design

This study was a 3-armed randomized controlled trial with intervention (executive function training app, self-guided CBT app, and waitlist control) as the group variable and time (baseline, postintervention, and follow-up) as the repeated measure. Participants were allocated to groups via block randomization [37], with block sizes of 6, 9, and 12, and a list created using Sealed Envelope (Sealed Envelope Ltd). The primary researcher (AM) had access to the allocation sequence and was not blinded to intervention assignment.

### Ethical Considerations

This study was approved by the University of Bath Psychology Research Ethics Committee (number: 22-049). Participants were presented with study information and researcher contact details in the event of any questions, prior to signing a consent form online. Participants were reimbursed £10 (approximately US $13) per hour for completing testing sessions in the study or were offered course credit if recruited through the university’s research participation scheme. Participants were provided with links to local and national mental health support charities after each testing session and as part of the debriefing process. No identifying information of participants was recorded in the final dataset, and participants provided consent for sharing of their anonymized data.

### Participants

Participants were eligible for inclusion if they were aged between 18 and 67 years (the state retirement age in the United Kingdom) and were either employed part-time or full-time or self-employed. Participants were included if they scored above 5 on the Patient Health Questionnaire-9 (PHQ-9) and above 4 on the Generalized Anxiety Disorder-7 (GAD-7), indicating clinically relevant mild symptoms of anxiety and depression [38]. Participants were ineligible if they were receiving talking therapy or were due to start talking therapy in the next 12 weeks. Computer literacy was assumed but was not an explicit inclusion criterion. A total of 240 participants were recruited via advertisements placed on social media and mental health charity research boards (eg, MQ Mental Health), research participation schemes (eg, SONA), the University of Bath’s community research participation panel, and Prolific Academic, as well as through word of mouth. Emails or Prolific messages were exchanged with applicants to protect against multiple or fraudulent submissions. After exclusion, our final sample included 228 participants (mean age 33.79, SD 11.50 years; female participants: 147/228, 64.5%).

### Study Groups

#### Waitlist Control

Participants assigned to the waitlist control group completed questionnaires and the executive function task at the same time points as those receiving the active interventions and were not instructed to download an app. Previous research has found that waitlist control conditions may exaggerate treatment effects in randomized controlled trials [39]; however, given the differing “active” components of our 2 intervention conditions, a waitlist control was considered as a pragmatic option for comparison.

#### Executive Function Training

Participants allocated to the executive function training intervention were provided with a subscription to NeuroNation, an executive function and brain training app, on their Android or iOS phone. The app presents participants with a selection of games to complete each day, aimed at improving attention, speed, and memory. The app provides psychoeducation and details how each game could improve functioning in everyday situations, such as conversations with loved ones. Each game takes around 120 seconds, with 10 games in each training session. Participants were instructed to complete a minimum of 21 sessions during the 28-day intervention period. In each session, participants completed gamified executive function tasks such as modified digit span or trail-making tasks. NeuroNation has been used in previous research investigating the effects of executive function training on working memory and broader cognitive function [17,40,41], with effect sizes ranging from *f*=0.23 [39] to *f*=0.30 [17]. Screenshots from the app are provided in Multimedia Appendix 1.

#### Self-Guided CBT

Participants allocated to the CBT intervention were provided with a subscription to Moodfit, a self-guided CBT and well-being app, on their Android or iOS phone. Moodfit was selected based on its layout, provision of core CBT techniques such as guided cognitive restructuring, and popularity. Participants were instructed to complete the mood journal each day and spend at least 10 minutes recording cognitive restructuring entries twice a day, for a minimum of 21 days over the 28-day intervention period. Screenshots from the Moodfit app are provided in Multimedia Appendix 1.

### Primary Outcomes

*Depressive symptoms* were measured with the PHQ-9 [42], which is a 9-item rating scale, with possible response options ranging from 0 (“not at all”) to 3 (“nearly every day”). Questions are related to general depressive symptoms experienced over the previous 2 weeks (eg, “Little interest or pleasure in doing things?”). Higher scores represent greater symptom severity. This measure was chosen owing to its reliability and validity in the general population [43], and it had a Cronbach α value of 0.82 in our sample at baseline.

*Anxiety symptoms* were measured with the GAD-7 [44], which is a 7-item rating scale, with possible response options ranging from 0 (“not at all”) to 3 (“nearly every day”). Questions are related to the frequency of anxiety symptoms over the past 2 weeks (eg, “Not being able to stop or control worrying?”). Higher scores represent greater symptom severity. This measure was chosen owing to its reliability and validity in the general population [45], and it had a Cronbach α value of 0.86 in our sample at baseline.

*Workplace well-being* was measured with the Utrecht Work Engagement Scale [46], which is a 9-item rating scale, with responses ranging from 1 (“never”) to 7 (“every day”). Questions are related to engagement and motivation at work (eg, “I am immersed in my work”). This scale has been found to have good reliability and validity in previous research [47], and it had a Cronbach α value of 0.93 in our sample at baseline.

*Working memory capacity* was assessed with the Operation Span (OSPAN) task [48]. Participants solved a set of simple arithmetic problems while simultaneously remembering a string of letters, with set sizes ranging from 3 to 7 letters. Set sizes were repeated thrice, for a total of 15, with the set order randomized by Inquisit 6 (Millisecond). Absolute scoring (the sum of all letters recalled in the correct position) was used, with higher OSPAN scores indicating greater working memory capacity.

### Secondary Outcomes

*Daily life stress* was measured with the Survey of Recent Life Experiences [49], which is a 41-item scale asking about the frequency of daily life stressors (eg, “being ignored” and “financial burdens”) over the past 4 weeks. Responses range from 1 (“not at all part of my life”) to 4 (“very much part of my life”), with higher scores indicating more stress over the past month. This scale has been shown to have good construct validity [50], and it had a Cronbach α value of 0.90 in our sample at baseline.

*Social workplace well-being* was measured with the Eudaimonic Workplace Wellbeing Scale [51], which is an 8-item, 5-point rating scale designed to measure the social dimension of well-being in a workplace context and general workplace well-being (eg, “I feel close to the people in my work environment”). Responses range from 1 (“strongly disagree”) to 5 (“strongly agree”), with higher scores indicating higher or more positive eudaimonic well-being in the context of the workplace. This scale has been shown to have good construct validity [51], and it had a Cronbach α value of 0.85 in our sample at baseline.

*General mental well-being* was measured with the Short Warwick and Edinburgh Mental Well-being Health Scale [52]. This is a 7-item, 5-point rating scale designed to measure mental well-being, with questions related to the frequency of positive emotions or behaviors over the past 2 weeks (eg, “I’ve been feeling optimistic about the future” and “I’ve been dealing with problems well”). This scale has been shown to have good construct validity [53], and it had a Cronbach α value of 0.81 in our sample at baseline.

*Workplace absence*, *stressful events*, and *behaviors* were measured via a series of binary response questions at each time point. Participants were asked whether they had experienced a stressful event at work in the last 7 days, taken time off work due to stress, worked outside of contracted hours or annual leave, or come in to work when they were ill in the past month.

*Intervention adherence* was measured via self-report as part of the postintervention testing session. Participants were asked if they had used the app on at least 21 days over the 4-week training period and asked about the number of sessions they thought they had completed.

### Procedure

Participants completed baseline, postintervention (4 weeks after baseline), and follow-up (12 weeks after baseline) testing sessions online. Participants were presented with an information sheet on the recruitment site (available with data) prior to signing a consent form online. After gaining informed consent, questionnaires were completed online using Qualtrics (Qualtrics, LLC) and QuestionPro (QuestionPro Inc), followed by the OSPAN task on Inquisit 6. Upon completion of their first testing session, participants were emailed a link revealing their training group and provided with links to download and register their app, if they were assigned to one of the active intervention groups.

### Statistical Analysis

All analyses were carried out using RStudio (Posit; see Multimedia Appendix 1 for packages and references). Missing data were assessed for randomness using the Little test [54] and found to be missing completely at random at both postintervention (168/226 complete participants; 26% missing; *χ*^2^_8_=6.94; *P*=.54) and follow-up (95/228 complete participants; 58% missing; *χ*^2^_8_=8.00; *P*=.33). Missingness was similar across groups. Simulation-based power analyses found that the smallest detectable postintervention interaction effect size with 80% power would be β=3.29, and this would increase to β=3.95 after attrition at follow-up.

In line with our preregistered analysis plan, mixed linear models were conducted to test hypotheses 1a-c. In all models, *participant* was set as a random effect and compared to an intercept-only model to determine if model fit was improved by its inclusion. Group (dummy coded with waitlist control set as the reference), time (dummy coded with baseline set as the reference), and group × time interactions were included as predictor variables. We also conducted regression models for postintervention and follow-up scores of depressive and anxiety symptoms and workplace well-being while adjusting for baseline scores. These results are reported in Multimedia Appendix 1. In all analyses, we controlled for multiple comparisons with Benjamini-Hochberg correction for the false discovery rate (FDR [55]) for each hypothesis separately (eg, hypothesis 1a, hypothesis 1b, and hypothesis 1c), in addition to related exploratory variables.

To test hypotheses 2a-c, mediation models determined whether intervention-related changes in depressive symptoms were mediated by changes in working memory capacity. Following information provided by Hayes and Rockwood [56], we used the causal step approach for each outcome variable, and the significance of the mediation was determined with the bootstrap method (5000 bootstrap samples).

### Exploratory Sensitivity Analysis

To explore any clinical relevance of our results, we conducted binomial logistic regression in a subsample (n=122) of participants who scored above 10 on the PHQ-9 and above 8 on the GAD-7 at baseline to explore the effect of intervention group on recovery and the minimal clinically important difference in depressive symptoms. Recovery, according to the National Health Service Improving Access to Psychological Therapies definition, is moving from caseness (PHQ-9 score above 10 or GAD-7 score above 8) to below caseness after treatment [57]. Given our subclinical population, the minimal clinically important difference was set as a 20% reduction in the PHQ-9 score [58,59] for this subsample.

### Exploratory Analysis

We explored the effect of the intervention group on general well-being and daily stress with linear models adjusted for baseline well-being and daily stress scores, respectively. We additionally explored the effect of training group on incidents of workplace stress, absenteeism, presenteeism, and working while on leave. Finally, we explored the relationships of baseline depression and anxiety with OSPAN task performance. These results are presented in Multimedia Appendix 1.

## Results

### Sample Characteristics

**Figure 1.**
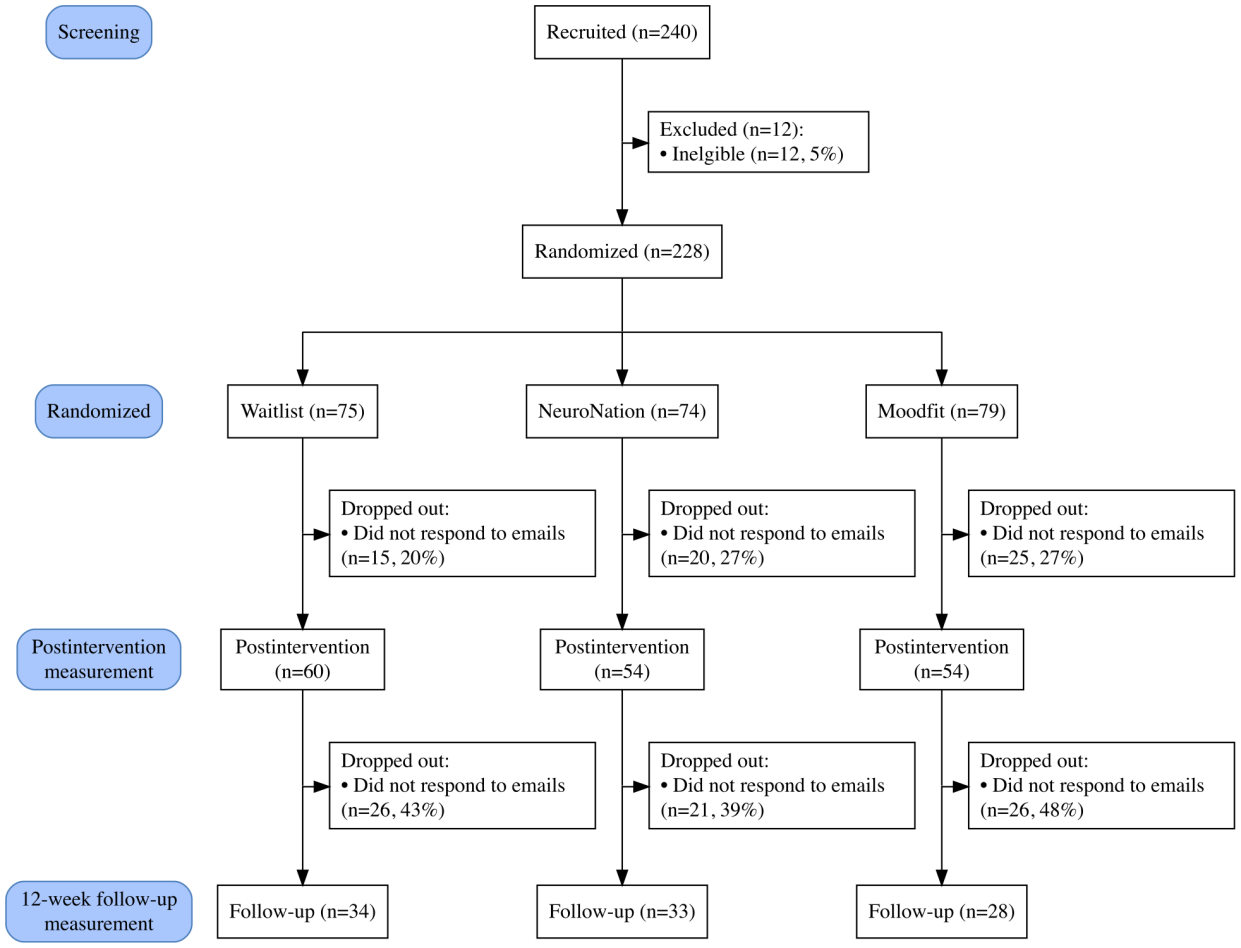
CONSORT diagram showing participant flow through the study.

Our sample included 228 participants (mean age 33.79, SD 11.50 years; range 18‐67 years; female participants: 147/228, 64.5%). The CONSORT (Consolidated Standards of Reporting Trials) checklist is presented in Checklist 1, and the CONSORT flow diagram is presented in Figure 1. No baseline differences in age (*F*_2,225_=0.01; *P*=.97) or gender (*χ*^2^_2_=0.28; *P*=.87) were found between the groups, with most participants being female, White, single, and an employee of a company (rather than self-employed; see Table 1). Most participants (155/228, 68.0%) had experienced at least one stressful event in the month leading up to their participation in the study, and 161 (71%) reported taking at least 1 day off work due to stress or mental health concerns. Posttraining self-reported intervention adherence was reasonable, with 89% (48/54) adherence to the NeuroNation (executive function training) app and 96% (52/54) adherence to the Moodfit (CBT-based) app.

**Table 1.** Sample characteristics at baseline.

Variable		Waitlist control group (n=75)	Executive function training (NeuroNation) group (n=74)	Self-guided CBT^a^ (Moodfit) group (n=79)
Age (years), mean (SD)		33.71 (12.44)	33.66 (11.65)	33.98 (10.65)
Female, n (%)		46 (61)	49 (65)	52 (66)
Ethnicity, n (%)				
Asian or British Asian		8 (11)	6 (8)	9 (11)
East Asian		0 (0)	3 (4)	2 (3)
Black/Black British		5 (7)	0 (0)	1 (1)
Mixed		3 (4)	1 (1)	2 (3)
White		45 (60)	53 (72)	56 (71)
Other		3 (4)	1 (1)	0 (0)
Did not answer		11 (14)	10 (14)	9 (11)
Marital status, n (%)				
Divorced		3 (4)	2 (3)	1 (1)
Married/living with partner		24 (32)	27 (36)	28 (35)
Single		30 (40)	30 (41)	34 (43)
Separated/widowed		3 (4)	2 (3)	2 (3)
Did not answer		15 (20)	13 (17)	14 (18)
Employment status, n (%)				
Employee		48 (64)	49 (66)	51 (65)
Management		5 (7)	7 (9)	6 (8)
Self-employed		5 (7)	4 (5)	8 (10)
Did not specify		17 (22)	14 (20)	14 (17)
Events experienced in the last month, n (%)				
Stressful event		51 (68)	48 (66)	56 (71)
Time off work due to stress		50 (67)	52 (71)	59 (75)
Training adherence (self-reported at posttraining; n=54)				
Completed >21 sessions, n (%)		—^b^	48 (89)	52 (96)
Sessions completed, mean (SD)		—	22.59 (11.39)	24.57 (10.95)

a CBT: cognitive behavioral therapy.

b Not applicable.

### Effects on Mental Health and Workplace Well-Being Outcomes

#### Overview

Descriptive statistics for all primary outcomes are presented in Table 2. The groups did not differ in terms of outcome measures at baseline.

**Table 2.** Results for the primary outcome variables.

Variable and time point	Waitlist control group (n=75), mean (SD)	Executive function training (NeuroNation) group (n=74), mean (SD)	Self-guided CBT^a^ (Moodfit) group (n=79), mean (SD)	*P* value^b^
Depressive symptoms (PHQ-9^c^)				
Baseline	10.08 (5.78)	10.03 (5.24)	9.96 (4.97)	.99
Postintervention	8.63 (5.64)	7.72 (5.37)	6.85 (5.42)	—^d^
Follow-up	8.86 (5.38)	7.52 (6.07)	8.29 (4.75)	—
Anxiety symptoms (GAD-7^e^)				
Baseline	9.11 (5.30)	9.41 (4.67)	9.15 (4.33)	.92
Postintervention	7.97 (4.85)	7.00 (4.35)	6.74 (4.17)	—
Follow-up	8.63 (5.57)	6.58 (4.91)	7.15 (4.48)	—
Workplace well-being (UWES-9^f^)				
Baseline	41.09 (14.38)	42.03 (11.33)	41.41 (11.38)	.90
Postintervention	38.97 (13.69)	43.80 (12.24)	43.81 (11.53)	—
Follow-up	40.31 (13.32)	43.81 (13.87)	43.47 (11.85)	—
Working memory capacity (OSPAN^g^)				
Baseline	40.30 (18.59)	36.11 (18.69)	40.87 (19.34)	.25
Postintervention	45.72 (17.99)	44.32 (20.00)	42.08 (17.91)	—
Follow-up	44.61 (19.56)	42.36 (21.64)	49.38 (16.57)	—

a CBT: cognitive behavioral therapy.

b Baseline differences were tested using 1-way ANOVA.

c PHQ-9: Patient Health Questionnaire-9.

d Not applicable.

e GAD-7: Generalized Anxiety Disorder-7.

f UWES-9: Utrecht Work Engagement Scale-9.

g OSPAN: Operation Span.

#### Effects of Executive Function Training or Self-Guided CBT on Depressive Symptoms

**Figure 2.**
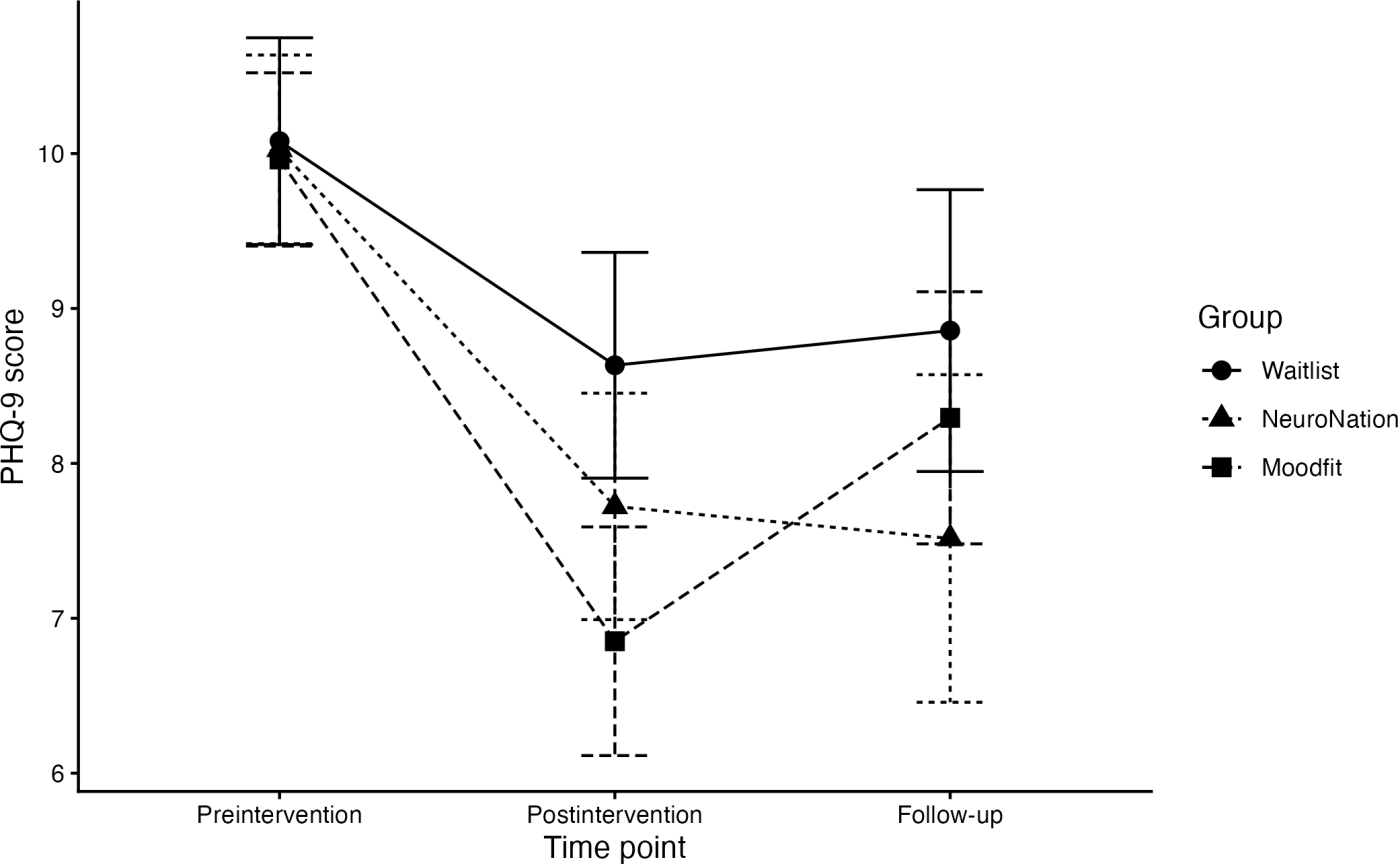
Change in depressive symptoms in each intervention group over time. PHQ-9: Patient Health Questionnaire-9.

Addressing hypothesis 1a, a mixed linear model testing the effects of executive function training or self-guided CBT on depressive symptoms (Table 3) found a main effect of time (*χ*²_2_=35.69; *P*<.001)*,* suggesting that depressive symptoms decreased in all groups across the study, though there was no group × time interaction (*χ*²_4_=7.63; *P*=.11). We found an effect of assignment to the NeuroNation group on depressive symptoms at the 12-week follow-up (*b*=−2.77, 95% CI −4.96 to −0.56; *P*=.02), suggesting an average reduction of 2.77 points in the PHQ-9 score compared with the finding in the waitlist control group, partially supporting hypothesis 1a (see Figure 2), though these effects did not survive Benjamini-Hochberg correction (adjusted *P*=.06). There was no effect of assignment to the self-guided CBT app at either time point.

**Table 3.** Parameter estimates for mixed linear models involving depressive symptoms (PHQ-9^a^), anxiety symptoms (GAD-7^b^), and workplace well-being (UWES-9^c^).

Variable		β	SE	95% CI	*P* value	Adjusted *P* value
Depressive symptoms (PHQ-9)						
Group		1.67	—^d^	—	.44	—
Waitlist control (reference)		—	—	—	—	—
NeuroNation		−0.15	0.87	−1.85 to 1.56	.87	—
Moodfit		0.09	0.87	−1.61 to 1.79	.92	—
Time		35.69	—	—	<.001^e^	—
Baseline (reference)		—	—	—	—	—
Postintervention		−1.32	0.64	−2.57 to −0.08	.04^e^	—
Follow-up		−0.30	0.79	−1.83 to 1.23	.71	—
Group × time		7.63	—	—	.11	—
NeuroNation × postintervention		−1.09	0.93	−2.89 to 0.72	.24	.24
Moodfit × postintervention		−1.51	0.92	−3.30 to 0.29	.10	.26
NeuroNation × follow-up		−2.77	1.13	−4.96 to −0.56	.02^e^	.06
Moodfit × follow-up		−1.56	1.12	−3.74 to 0.62	.16	.22
Anxiety symptoms (GAD-7)						
Group		2.01	—	—	.37	—
Waitlist control (reference)		—	—	—	—	—
NeuroNation		0.22	0.77	−1.28 to 1.72	.78	—
Moodfit		−0.21	0.76	−1.70 to 1.28	.78	—
Time		41.16	—	—	<.001^e^	—
Baseline (reference)		—	—	—	—	—
Postintervention		−1.15	0.55	−2.22 to −0.08	.04^e^	—
Follow-up		−0.07	0.67	−1.39 to 1.24	.91	—
Group × time		9.58	—	—	.048^e^	—
NeuroNation × postintervention		−1.34	0.79	−2.88 to 0.21	.09	.12
Moodfit × postintervention		−1.24	0.79	−2.78 to 0.29	.12	.12
NeuroNation × follow-up		−2.79	0.97	−4.68 to −0.91	.004^e^	.02^f^
Moodfit × follow-up		−1.86	0.96	−3.73 to 0.01	.054	.11
Workplace well-being (UWES-9)						
Group		1.34	—	—	.51	—
Waitlist control (reference)		—	—	—	—	—
NeuroNation		0.67	2.10	−3.43 to 4.76	.75	—
Moodfit		−0.11	2.09	−4.19 to 3.97	.96	—
Time		1.04	—	—	.59	—
Baseline (reference)		—	—	—	—	—
Postintervention		−1.43	1.10	−3.58 to 0.72	.20	—
Follow-up		−2.23	1.36	−4.88 to 0.43	.10	—
Group × time		8.64	—	—	.07	—
NeuroNation × postintervention		2.70	1.61	−0.43 to 5.83	.09	.09
Moodfit × postintervention		3.72	1.59	0.62 to 6.82	.02^e^	.046^f^
NeuroNation × follow-up		3.67	1.98	−0.17 to 7.51	.06	.09
Moodfit × follow-up		4.46	1.94	0.67 to 8.24	.02^e^	.046^f^

a PHQ-9: Patient Health Questionnaire-9.

b GAD-7: Generalized Anxiety Disorder-7.

c UWES-9: Utrecht Work Engagement Scale-9.

d Not applicable.

e Significant (*P*<.05).

f Result survived Benjamini-Hochberg correction for the false discovery rate.

In post hoc sensitivity analyses of recovery and minimal clinically important differences (Table 4), we found that those assigned to the executive function training and self-guided CBT groups were 4.77 times and 3.82 times more likely than the waitlist control group to no longer meet caseness criteria after the intervention, respectively. When assessing minimal clinically important differences, the executive function training and self-guided CBT groups were 3.54 and 3.52 times more likely than the waitlist control group to experience a clinically meaningful improvement in depressive symptoms, respectively (Table 4). These results survived FDR correction (all *P*<.05; detailed results are presented in Multimedia Appendix 1). However, there was no effect of group assignment at the 12-week follow-up testing session.

**Table 4.** Results for binomial logistic regression models investigating the effect of training on the likelihood of recovery and experiencing a minimal clinically meaningful reduction in symptoms.

Model	Time point	Executive function training (NeuroNation) group (reference: waitlist control group)		Self-guided CBT^a^ (Moodfit) group (reference: waitlist control group)	
		Exp(*b*), value (95% CI)	*P* value	Exp(*b*), value (95% CI)	*P* value
PHQ-9^b^, recovery	4 weeks	4.77 (1.59-14.30)	.005^c^^,^^d^	3.82 (1.25-11.69)	.02^c^^,^^d^
PHQ-9, recovery	12 weeks	5.33 (1.28-22.19)	.02^c^^,^^d^	2.00 (0.49-8.24)	.34
PHQ-9, MCID^e^	4 weeks	3.54 (1.19-10.50)	.02^c^^,^^d^	3.66 (1.13-10.88)	.03^c^^,^^d^
PHQ-9, MCID	12 weeks	1.78 (0.44-7.18)	.42	0.83 (0.21-3.35)	.80
GAD-7^f^, recovery	4 weeks	3.75 (1.38-10.17)	.009^c^^,^^d^	1.96 (1.58-12.14)	.005^c^^,^^d^
GAD-7, recovery	12 weeks	3.18 (0.72-12.94)	.11	3.50 (0.85-14.41)	.08
GAD-7, MCID	4 weeks	1.84 (0.69-4.87)	.22	3.13 (1.07-9.09)	.04^c^
GAD-7, MCID	12 weeks	1.33 (0.38-4.73)	.66	2.33 (0.62-8.82)	.21

a CBT: cognitive behavioral therapy.

b PHQ-9: Patient Health Questionnaire-9.

c Significant (*P*<.05).

d Result survived Benjamini-Hochberg correction for the false discovery rate.

e MCID: minimal clinically important difference.

f GAD-7: Generalized Anxiety Disorder-7.

With regard to hypothesis 2a, the causal step mediation model found no mediating effect of change in executive function on intervention-related changes in depressive symptoms from pre- to postintervention (*b*=0.02, 95% CI −0.16 to 0.26; *P*=.84; Table 5). Taken together, these results suggest that assignment to the executive function training group reduced depressive symptoms when compared with assignment to the waitlist control group, partially supporting hypothesis 1a, though there was no effect of assignment to the self-guided CBT group in our preregistered analyses. Exploratory analyses suggested that among those with moderate symptoms of depression, assignment to an intervention group resulted in improved outcomes. However, these improvements were not mediated by changes in executive function, and thus, the findings fail to support hypothesis 2a.

**Table 5.** Parameter estimates for mediation models.

Variable		β	95% CI	*P* value
Δ^a^OSPAN^b^				
NeuroNation		1.29	−6.40 to 8.99	.74
Moodfit		−7.16	−14.85 to 0.54	.07
ΔPHQ-9^c^				
NeuroNation		−1.30	−3.40 to 0.79	.22
Moodfit		−1.32	−3.44 to 0.80	.22
ΔOSPAN		0.01	−0.03 to 0.06	.51
Indirect effect		0.02	−0.17 to 0.26	.84
ΔGAD-7^d^				
NeuroNation		−1.49	−3.24 to 0.25	.09
Moodfit		−0.86	−2.61 to 0.90	.34
ΔOSPAN		0.03	−0.00 to 0.07	.07
Indirect effect		0.04	−0.20 to 0.31	.71
ΔUWES-9^e^				
NeuroNation		2.23	−1.16 to 5.61	.20
Moodfit		3.52	0.10 to 6.94	.04^f^
ΔOSPAN		−0.05	−0.12 to 0.02	.17
Indirect effect		−0.06	−0.52 to 0.25	.70

a Change scores are calculated from pre- to postintervention.

b OSPAN: Operation Span.

c PHQ-9: Patient Health Questionnaire-9.

d GAD-7: Generalized Anxiety Disorder-7.

e UWES-9: Utrecht Work Engagement Scale-9.

f Significant (*P*<.05).

#### Effects of Executive Function Training or Self-Guided CBT on Anxiety Symptoms

**Figure 3.**
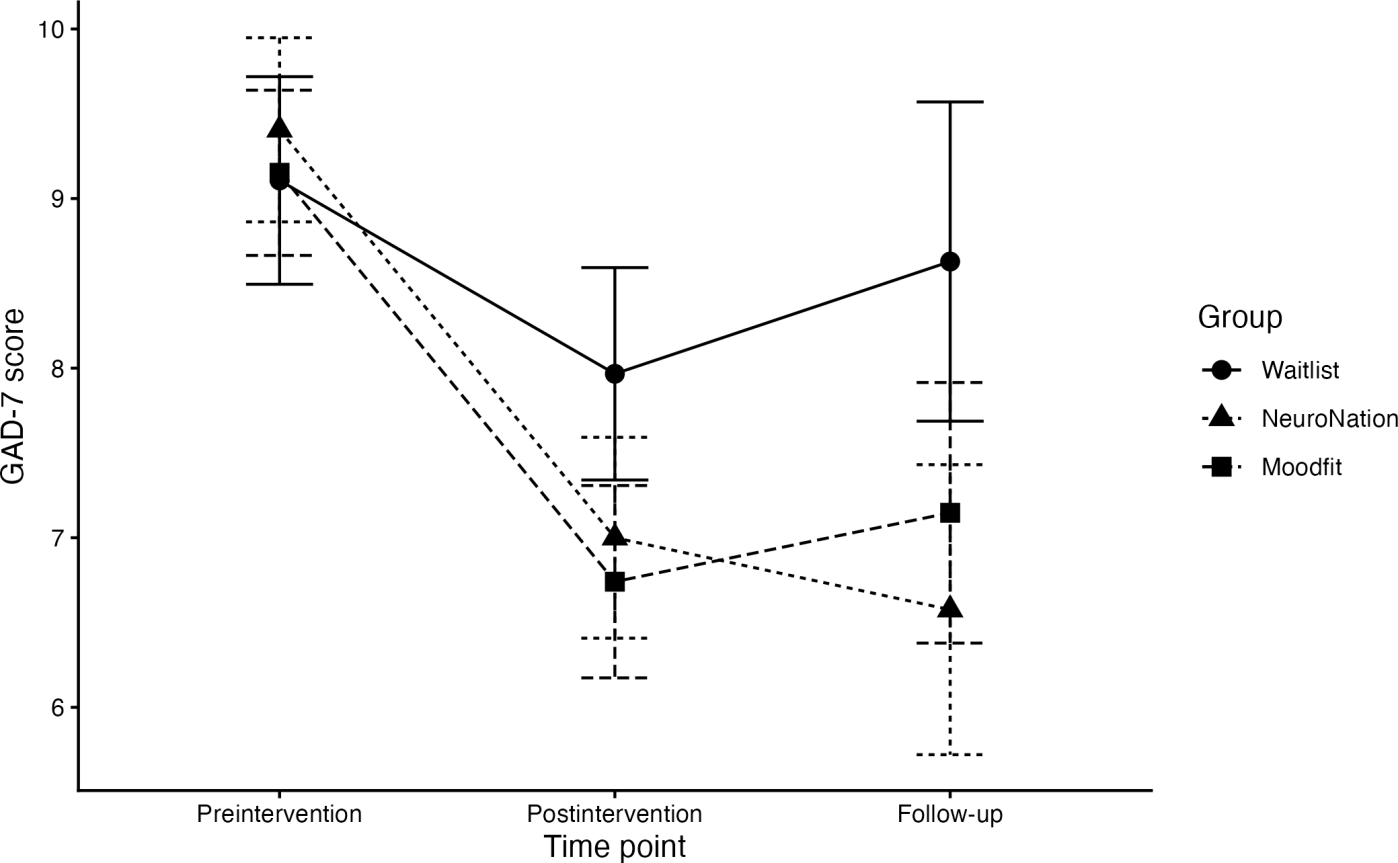
Change in anxiety symptoms in each intervention group over time. GAD-7: Generalized Anxiety Disorder-7.

Addressing hypothesis 1b, the mixed linear model testing the effects of executive function training or self-guided CBT on anxiety symptoms (Table 3) found a main effect of time (*χ*²_2_=41.16; *P*<.001)*,* suggesting that anxiety symptoms decreased in all groups across the study, and a group × time interaction was observed (*χ*²_4_=9.58; *P*=.048). We found an effect of assignment to the executive function training group on anxiety symptoms at follow-up (*b*=−2.79, 95% CI −4.68 to −0.91; *P*=.004), suggesting an average reduction of 2.79 points in the GAD-7 score compared with the finding in the waitlist control group, and this survived FDR correction (adjusted *P*=.02), partially supporting hypothesis 1b (Figure 3). There was weak evidence that participants assigned to the self-guided CBT group demonstrated lower anxiety scores at follow-up compared with the waitlist control group (*b*=−1.86, 95% CI −3.73 to 0.01; *P*=.054), though CIs crossed the null value.

Our post hoc sensitivity analyses of recovery and minimal clinically important differences (Table 4) found that the executive function training and self-guided CBT groups were 3.75 and 4.38 times more likely than the waitlist control group to no longer meet caseness, respectively. Additionally, those allocated to the self-guided CBT group were 3.13 times more likely to experience a clinically meaningful reduction in symptoms, though this finding did not survive correction. No other effects met the significance criteria.

With regard to hypothesis 2b, the causal step mediation model found no mediating effect of change in executive function on training-related changes in anxiety symptoms from pre- to postintervention (*b*=0.04, 95% CI −0.20 to 0.31; *P*=.71; Table 5). These results suggest that assignment to the executive function training group improved anxiety symptoms when compared with assignment to the waitlist control group, partially supporting hypothesis 1b, with weak evidence of an effect in the self-guided CBT group in our preregistered analyses. As with depressive symptoms, our exploratory analyses suggested that assignment to a training group increased the probability of no longer meeting caseness after the intervention, with evidence suggesting that those completing self-guided CBT were more likely to experience a meaningful reduction in symptoms compared with waitlist controls. However, this was not mediated by changes in executive function, and thus, the findings fail to support hypothesis 2b.

#### Effects of Executive Function Training or Self-Guided CBT on Workplace Well-Being

**Figure 4.**
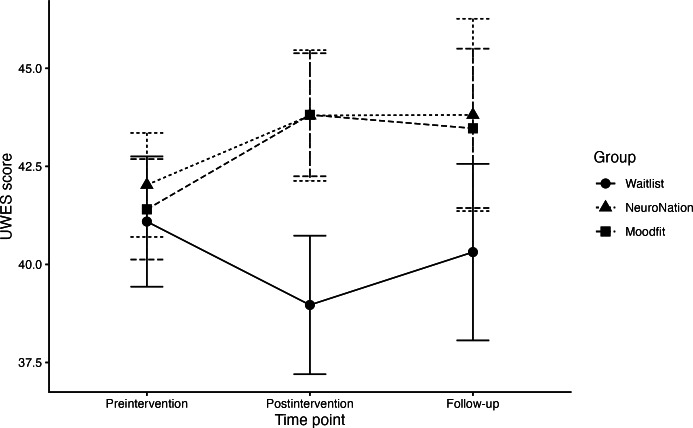
Change in workplace well-being scores in each intervention group over time. UWES: Utrecht Work Engagement Scale.

In the mixed linear model testing the effects of executive function training or self-guided CBT on workplace well-being (hypothesis 1c), we found no main effect of time and no overall group × time interaction, with all parameters presented in Table 3. We found an effect of assignment to the self-guided CBT group at postintervention (*b*=3.72, 95% CI 0.62-6.82; *P*=.02) and follow-up (*b*=4.46, 95% CI 0.67-8.24; *P*=.02), suggesting an improvement of almost 4 points in workplace well-being compared with the finding in the waitlist control group, partially supporting hypothesis 1c (Figure 4). These results survived correction (both adjusted *P*<.05); however, CIs in the executive function training group crossed the null value (Table 3).

Regarding hypothesis 2c, the causal step mediation model found no mediating effect of change in executive function on training-related changes in workplace well-being from pre- to postintervention (*b=*−0.06, 95% CI −0.52 to 0.25; *P*=.70; Table 5). These results suggest that assignment to the self-guided CBT group benefited workplace well-being when compared with assignment to the waitlist control group, though no benefit of assignment to the executive function training group was found, partially supporting hypothesis 1c. However, this was not mediated by changes in executive function, and thus, the findings fail to support hypothesis 2c.

### Planned Exploratory Analyses: Training Effects on General Well-Being, Social Workplace Well-Being, and Daily Stress

Linear regression models found evidence for an effect of the intervention on daily life stresses, consistent with our expectations (Table 6). At postintervention, participants assigned to the executive function training group (*b*=−4.96, 95% CI −9.43 to −0.49; *P*=.03) and those assigned to the self-guided CBT group (*b*=−7.81, 95% CI −12.24 to −3.38; *P*=.001) reported fewer daily life stresses compared with those assigned to the waitlist control group. These effects were sustained at follow-up for both the executive function training group (*b*=−7.39, 95% CI −12.81 to −1.90; *P*=.009) and the self-guided CBT group (*b*=−5.58, 95% CI −10.98 to −0.18; *P*=.045).

**Table 6.** Parameter estimates for mixed linear models involving stressful life events (SRLE^a^), general well-being (SWEMWBS^b^), and social workplace well-being (EWWS^c^).

Variable		β	SE	95% CI	*P* value	Adjusted *P* value
Stressful life events (SRLE)						
Group		9.41	—^d^	—	.009^e^	—
Waitlist control (reference)		—	—	—	—	—
NeuroNation		−5.24	2.82	−10.74 to 0.25	.06	—
Moodfit		−1.00	2.80	−6.47 to 4.47	.72	—
Time		36.09	—	—	<.001^e^	—
Baseline (reference)		—	—	—	—	—
Postintervention		−1.24	1.58	−4.31 to 1.83	.43	—
Follow-up		−0.16	1.95	−3.95 to 3.63	.94	—
Group × time		15.91	—	—	.003^e^	—
NeuroNation × postintervention		−4.96	2.30	−9.43 to −0.49	.03^e^	.04^f^
Moodfit × postintervention		−7.81	2.28	−12.24 to −3.38	.001^e^	.004^f^
NeuroNation × follow-up		−7.36	2.80	−12.81 to −1.90	.009^e^	.02^f^
Moodfit × follow-up		−5.58	2.78	−10.98 to −0.18	.045^e^	.045^f^
General well-being (SWEMWBS)						
Group		1.20	—	—	.55	—
Waitlist control (reference)		—	—	—	—	—
NeuroNation		−0.54	0.68	−1.87 to 0.78	.43	—
Moodfit		−0.59	0.68	−1.91 to 0.72	.38	—
Time		15.37	—	—	<.001^e^	—
Baseline (reference)		—	—	—	—	—
Postintervention		−0.55	0.47	−1.48 to 0.37	.25	—
Follow-up		0.01	0.58	−1.13 to 1.14	.99	—
Group × time		20.52	—	—	<.001^e^	—
NeuroNation × postintervention		2.48	0.69	1.14 to 3.83	<.001^e^	.002^f^
Moodfit × postintervention		2.32	0.68	0.98 to 3.65	.001^e^	.002^f^
NeuroNation × follow-up		2.33	0.84	0.69 to 3.96	.006^e^	.008^f^
Moodfit × follow-up		0.72	0.83	−0.90 to 2.34	.39	.39
Social workplace well-being (EWWS)						
Group		1.34	—	—	.51	—
Waitlist control (reference)		—	—	—	—	—
NeuroNation		0.56	1.07	−1.53 to 2.65	.60	—
Moodfit		0.86	1.07	−1.23 to 2.94	.42	—
Time		0.39	—	—	.82	—
Baseline (reference)		—	—	—	—	—
Postintervention		−0.01	0.65	−1.28 to 1.26	.99	—
Follow-up		−0.43	0.81	−2.00 to 1.14	.59	—
Group × time		2.83	—	—	.59	—
NeuroNation × postintervention		0.74	0.95	−1.11 to 2.60	.44	.87
Moodfit × postintervention		0.07	0.94	−1.76 to 1.91	.94	.99
NeuroNation × follow-up		1.68	1.16	−0.58 to 3.94	.15	.60
Moodfit × follow-up		−0.01	1.15	−2.25 to 2.22	.99	.99

a SRLE: Survey of Recent Life Experiences.

b SWEMWBS: Short Warwick and Edinburgh Mental Well-being Health Scale.

c EWWS: Eudaimonic Workplace Wellbeing Scale.

d Not applicable.

e Significant (*P*<.05).

f Result survived Benjamini-Hochberg correction for the false discovery rate.

There was also evidence that assignment to the executive function training group (*b*=2.48, 95% CI 1.14-3.83; *P*<.001) and assignment to the self-guided CBT group (*b*=2.32, 95% CI 0.98-3.65; *P*=.001) led to increases in postintervention well-being scores compared with assignment to the waitlist control group. This effect was sustained in the executive function training group (*b*=2.33, 95% CI 0.69-3.96; *P*=.001) but not in the self-guided CBT group at the 12-week follow-up. The results survived FDR correction. We found no effect of the intervention group on social workplace well-being.

### Unplanned Exploratory Analyses

In our unplanned exploratory analyses, we found no relationship between OSPAN task performance and depressive or anxiety symptoms. Additionally, we found no intervention effects on reported absenteeism, presenteeism, or working while on leave. The complete results are presented in Multimedia Appendix 1.

## Discussion

### Principal Findings

This study investigated the effectiveness of app-based executive function training and self-guided CBT for improving mental health and workplace well-being in a high-risk, working population of adults. It also examined whether improvements in executive function mediated any intervention-related improvements that were seen. Our hypotheses were partially supported, with mental health and well-being outcomes showing improvements in the active intervention groups compared with the waitlist control group, though contrary to expectations, changes in executive function were not found to mediate symptom improvement, even in the executive function training group.

### Effectiveness of App-Based Interventions for Mental Health and Workplace Well-Being

Partially supporting our first hypothesis, those assigned to the executive function training group reported significantly lower depressive and anxiety symptoms at the 12-week follow-up testing session compared with those assigned to the waitlist control group, though the effect for depression did not survive FDR correction. In our exploratory analysis focusing on above-threshold symptoms of depression and anxiety (scores above 10 on the PHQ-9 and above 8 on the GAD-7), we found that participants assigned to the executive function training group were less likely to report depressive and anxiety symptoms above those thresholds after the intervention and were more likely to experience a clinically meaningful (20%) reduction in depressive symptoms at postintervention compared with those assigned to the waitlist control group. Contrary to our hypotheses, there was no difference in workplace well-being between participants assigned to the executive function training group and those assigned to the waitlist control group. Exploratory analysis of our preregistered secondary outcome variables showed improvements in general well-being and lower levels of daily stress, with the effects sustained at follow-up. Taken together, these results suggest that executive function training may be effective in managing symptoms of depression and anxiety in a sample of individuals with mild-to-moderate symptoms, as well as in improving well-being, though this does not necessarily translate to changes in workplace behavior. This is consistent with previous reviews reporting that executive function training reduces depressive and anxiety symptoms (eg, [16]). Additionally, though we conducted and interpreted our results in accordance with our preregistered analysis plan, our omnibus interaction test was not significant for depressive symptoms, and thus, we suggest cautious interpretation of these findings.

Contrary to our hypotheses, the self-guided CBT group did not report significantly lower depressive symptoms compared with the waitlist control group at either the postintervention or follow-up time points. While there was weak evidence to suggest an effect on anxiety at the follow-up measurement, the CIs crossed the null value. In our exploratory analyses, participants assigned to the self-guided CBT group were more likely to experience both recovery from and meaningful reductions in depressive and anxiety symptoms after the intervention. Participants completing the self-guided CBT approach reported significant improvements in workplace well-being, improvements in general well-being, and reductions in daily stress after the intervention, with effects in workplace well-being and stress models being sustained at follow-up. This suggests that the app is effective at managing well-being, but effects may be more limited when applied to mental health outcomes. This is consistent with previous work showing that digital interventions with additional therapist support outperform self-guided programs [60]. On the other hand, it may simply reflect that the Moodfit app included techniques other than those derived from CBT (eg, mindfulness). While participants were instructed to complete the CBT techniques included in the app, there was no control over which modules participants completed. As a full course of CBT was not built into the app chosen and given that guided app-based CBT has previously been found to improve mental health symptoms [26], the unstructured nature of this program may be suitable for managing well-being, while mental health symptom management may require fidelity to established treatment protocols.

### Executive Function as a Mechanism of Action

Contrary to our hypotheses, we found no evidence that improvements in executive function mediated treatment response. This is consistent with previous research questioning whether executive function training transfers to untrained executive function tasks or unrelated outcomes (eg, mood) [7,61], as well as research reporting that improvements in neurocognitive functioning more broadly do not appear to mediate the effects of digital CBT for anxiety [62]. We propose 2 potential explanations for the lack of mediation effects. First, working memory, as measured by the OSPAN task, improved in all groups, suggesting practice effects. Second, while the task seems well-suited to assess executive function, it may be insufficient in isolation. The OSPAN task engages both working memory capacity and cognitive control by requiring individuals to hold task-relevant information in working memory and effectively shift attention when needed, for example, from completing a math problem to storing a new letter in working memory. However, with previous research suggesting that depression and anxiety are characterized by domain-specific deficits in executive function, such as inhibition and updating [63,64], a wider variety of tasks may need to be used to capture the full range of relevant executive function processes.

### Strengths and Limitations

This study had several strengths. First, the inclusion of 2 active groups reduced the likelihood of our findings being solely due to expectancy effects, given the improvement found in the executive function training group compared with the waitlist control group. Additionally, this study measured both positive (eg, well-being) and negative (eg, depressive symptoms) outcomes alongside more applied or “real-world” outcomes (eg, workplace absence), allowing us to make inferences about the effectiveness of the apps tested and app-based interventions in general. Furthermore, both intervention groups self-reported high adherence rates. While previous research has found app-based interventions and online research to suffer from poor adherence [27], we found adherence rates of >80% for both groups. The high adherence may reflect the low time investment or cognitive effort required to use the Moodfit app and the benefits of the visually engaging, gamified NeuroNation app. As gamification is believed to improve the rate of adherence to otherwise dull but cognitively demanding tasks [34], the NeuroNation app was chosen to increase adherence to the protocol. However, usage data were not collected from either app. This lack of objective measurement of intervention adherence is a significant limitation of the study, and as this measure was added after data collection had commenced, the self-reported data for app usage are incomplete. Given that self-reported measures of behavior can lead to social desirability biases, which may lead to participants overreporting the number of completed sessions [65], gaining access to objective measures of adherence (eg, app usage data) would have strengthened our findings.

Additionally, we offered incentives to participate in the study, and this approach has been found to improve adherence to training programs [66]. This may have artificially inflated the effects, and in real-world use, where engagement with a training app is not incentivized, the reported effects may be diluted or not apparent. We also recruited participants through different methods (mental health research sites, Prolific Academic, and word of mouth), and thus, participants may have shown variations in motivation or intervention adherence. Though research has found Prolific Academic to provide higher quality data on behavioral tasks than other recruitment sites [67], there is limited research looking at the effect this may have on trials such as our trial.

We had little control over the content participants engaged with while using the apps, and app versions were not frozen for the duration of the trial. While this was not an issue for participants assigned to the NeuroNation app, which delivers a variety of executive function tasks in each training session, those assigned to Moodfit were faced with a “toolbox” style intervention, the contents of which may have changed (though CBT techniques were unchanged throughout). Although participants were instructed on which tasks to complete, the lack of control over content and the adoption of adherence checks only after the intervention period constitute limitations. Future research should use more controlled digital CBT interventions and endeavor to acquire usage data as a more robust measure of intervention adherence. Additionally, given that attrition reduced statistical power, especially at follow-up, we may have been unable to detect more subtle effects. Finally, the use of the OSPAN task as a lone measure of executive function may have influenced the results, especially given the lack of a relationship with either of our mental health outcome measures, challenging a key assumption of this study.

### Implications

This study has several implications. First, app-based executive function training appears to be an effective intervention for people with mild-to-moderate symptoms of depression and anxiety over 4- and 12-week periods. Given the improvement in general well-being and reduction in experienced daily stress, executive function training apps may be appropriate for well-being support, though there appeared to be no benefit for workplace well-being specifically. If our results are replicated in clinically relevant samples (eg, people currently awaiting treatment), such apps could be considered viable options in supporting mental health services.

The effectiveness of an app-based CBT technique, such as that delivered by Moodfit, is less clear. Improvements in daily stress and well-being suggest some utility in terms of well-being support as part of workplace well-being initiatives or otherwise, and though there is exploratory evidence supporting applications for clinical outcomes, these did not meet preregistered significance thresholds. Given previous work showing that human guidance improves adherence to digital interventions [68] and showing the positive effects of app-based CBT with therapist guidance and strict adherence to CBT protocols in clinical and nonclinical settings [26], future research may wish to select an app that delivers techniques in a structured manner, in alignment with CBT manuals (eg, [69]), as opposed to the general approach adopted by the app in this study.

Finally, our results raise questions about the role of executive function as a common pathway through which symptoms improve. Although the executive function training group experienced meaningful reductions in self-reported symptoms and increases in well-being, these were not accompanied by meaningful improvements in executive function. This may be due to the choice of executive function task, and thus, further research using a variety of tasks to capture different facets of executive function may be warranted. Additionally, deficits in executive function may only become apparent in daily life when people are operating at or near capacity (eg, balancing the stress of several projects at work and at home); therefore, adjusting tasks to imitate this level of cognitive load may improve the validity of the measure.

### Conclusion

This study set out to investigate the effectiveness of app-based executive function training and self-guided CBT for reducing depressive and anxiety symptoms and enhancing well-being in an “at-risk” working population. It also examined whether changes in executive function mediated symptom improvement. We found that executive function training reduced symptoms of depression and anxiety immediately after the intervention period and at a 12-week follow-up. We also found that a self-guided CBT app improved well-being after the intervention period and at follow-up, though no reductions in depressive or anxiety symptoms were observed, contrary to our expectations. Finally, there was no evidence that improvements in executive function mediated the effects of either intervention. These results suggest that executive function training may be an effective method for managing mild-to-moderate symptoms of depression and anxiety when waiting for active treatment, while self-guided CBT apps may be suitable for managing and improving well-being both in the workplace and in general. Future research should measure a wider variety of executive functions when investigating the mechanisms of action of psychological treatments such as executive function training.

## Supplementary material

10.2196/91564 Multimedia Appendix 1 Additional materials to support the study.

10.2196/91564 Checklist 1 CONSORT checklist.

## Abbreviations

CBT: cognitive behavioral therapy
CONSORT: Consolidated Standards of Reporting Trials
FDR: false discovery rate
GAD-7: Generalized Anxiety Disorder-7
OSF: Open Science Framework
OSPAN: Operation Span
PHQ-9: Patient Health Questionnaire-9
