# Correction: The Effectiveness and Mechanisms of Action of App-Based Interventions for Improving Mental Health and Workplace Well-Being: Randomized Controlled Trial

**DOI:** 10.2196/105585

**Published:** 2026-07-31

**Authors:** Alexander MacLellan, Graeme Fairchild, Katherine S Button

**Affiliations:** 1 Department of Psychology, University of Bath, Claverton Down, Bath, England, BA2 7AY, United Kingdom, 44 01225 388388

In “The Effectiveness and Mechanisms of Action of App-Based Interventions for Improving Mental Health and Workplace Well-Being: Randomized Controlled Trial” [1], the authors noted one error.

Reference 36 linking to the study dataset was incorrectly published as:

Dataset for “Investigating the effectiveness of digital cognitive training at improving mental health and social engagement in an ‘at risk’ population”. University of Bath Research Data Archive. 2025. URL: https://researchdata.bath.ac.uk/1491/ [Accessed 2026-04-10]

The reference has been corrected to:

Maclellan A, Fairchild G, Button K. Dataset for “The Effectiveness and Mechanisms of Action of App-based Interventions for Improving Mental Health and Workplace Well-Being: Randomized Controlled Trial.” University of Bath Research Data Archive; 2026. URL: https://doi.org/10.15125/BATH-01661 [Accessed 2026-07-20]

The correction will appear in the online version of the paper on the JMIR Publications website, together with the publication of this correction notice. Because this was made after submission to PubMed, PubMed Central, and other full-text repositories, the corrected article has also been resubmitted to those repositories.
