# Supplementary material for: The Effectiveness and Mechanisms of Action of App-Based Interventions for Improving Mental Health and Workplace Well-Being: Randomized Controlled Trial
Source: JMIR Ment Health. 2026 Apr 27;13:e91564. doi: 10.2196/91564 (PMC13119392; doi:10.2196/91564)
Supplement: Multimedia Appendix 1 [file mental-v13-e91564-s001.docx]

# Multimedia Appendix 1

Contents

[Multimedia Appendix 1 1](#_Toc227145578)

[Sample Size Estimation 1](#_Toc227145579)

[OSPAN Task Full Details 2](#_Toc227145580)

[Training App Screenshots 3](#_Toc227145581)

[1. Neuronation 3](#_Toc227145582)

[2. Moodfit 4](#_Toc227145583)

[R Package Citations 4](#_Toc227145584)

[Supplementary Analyses 10](#_Toc227145585)

[3. FDR adjusted p values for sensitivity analyses 10](#_Toc227145586)

[4. Results of primary outcome regression analyses with post-training or follow-up score as the outcome variable, controlling for baseline score 10](#_Toc227145587)

[5. Results of regression models of proportion of letters against depressive symptoms at each set size 11](#_Toc227145588)

[6. Results of regression models of proportion of letters against anxious symptoms at each set size 11](#_Toc227145589)

[7. Coefficients for logistic regressions of intervention effects on applied workplace outcomes 12](#_Toc227145590)

Sample Size Estimation

To determine our predicted sample size, we conducted a G*Power 3.1 (Faul et al., 2007) for a one-way ANOVA model, which is analogous to our predicted analysis plan of a regression adjusted for baseline differences. As suggested by Lakens (2023), we have powered this study to detect the smallest effect size of interest (0.44), taken from a recent meta-analysis (Motter et al., 2016). This resulted in a target sample size of 228 to achieve 80% power (76 per group). This was further supported by 2000 Monte Carlo simulations of this ANOVA using the ShinyApp R program (Lakens & Caldwell, 2019), which finds equal group sizes of 75 would be sufficient to achieve 80.15% power. This is similar in sample size to other 3-arm randomised controlled trials investigating depression and internet delivered CBT (e.g. Gold et al. 2023, 279 participants).

Faul F, Erdfelder E, Lang AG, Buchner A. G*Power 3: a flexible statistical power analysis program for the social, behavioral, and biomedical sciences. Behav Res Methods. 2007;39(2):175-191. doi:10.3758/BF03193146

Gold SM, et al. Internet-delivered cognitive behavioural therapy programme to reduce depressive symptoms in patients with multiple sclerosis: a multicentre, randomised, controlled, phase 3 trial. Lancet Digit Health. 2023;5(10):e668-e678. doi:10.1016/S2589-7500(23)00153-7

Lakens D. Sample size justification. Collabra Psychol. 2022;8(1):33267. doi:10.1525/collabra.33267

Lakens D, Caldwell AR. Simulation-based power analysis for factorial ANOVA designs. Computer software. 2019.

Motter JN, Pimontel MA, Rindskopf D, Devanand DP, Doraiswamy PM, Sneed JR. Computerized cognitive training and functional recovery in major depressive disorder: a meta-analysis. J Affect Disord. 2016;189:184-191. doi:10.1016/j.jad.2015.09.022

OSPAN Task Full Details

During the OSPAN task, participants solved a set of simple arithmetic problems whilst simultaneously remembering a string of letters. At the end of each set participants had to recall the letters in the correct order. For example, a set of 3 may be:

2 * 6 = ? – H

8 – 4 = ? – T

12 / 2 = ? – B

Set sizes range from 3 to 7 letters, with participants recalling the target letters in the correct order from a 3 x 4 letter matrix. Participants were given 5000ms to solve arithmetic problems before the trial failed and they were presented with the letter. Letters were shown for 1000ms and followed by a 500ms inter-trial interval. There was no time limit to recall letters. Between sets participants were shown performance feedback for both arithmetic and letter scores, with their arithmetic accuracy displayed in red in the top right corner of their screen. Each set size was repeated three times, for a total of 15, with set order randomised by Inquisit 6. Absolute scoring was used, being the sum of all letters recalled in the correct position, e.g. if the participant had entered HTB when shown the letters above, they would receive a score of three, whereas if they entered HBT they would score one. Higher OSPAN scores indicate greater working memory capacity.

## Training App Screenshots

### Neuronation


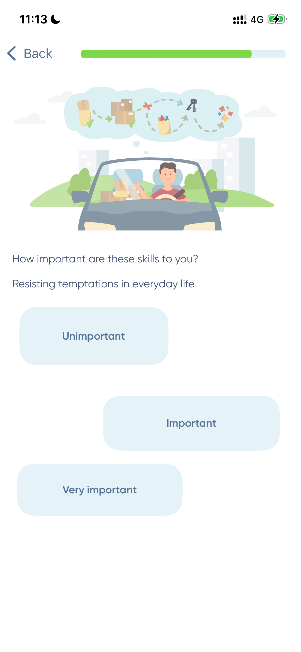

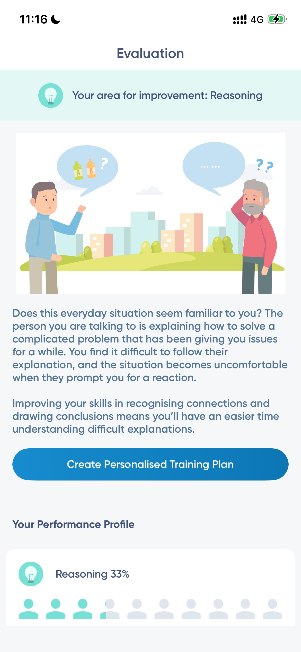

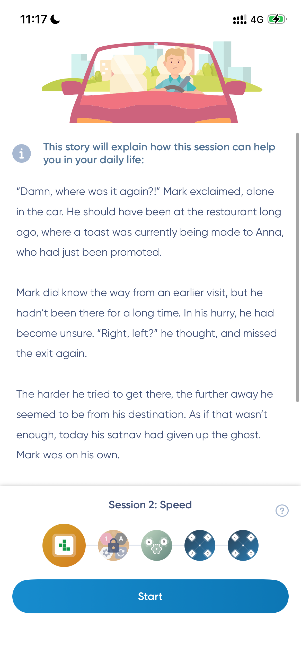

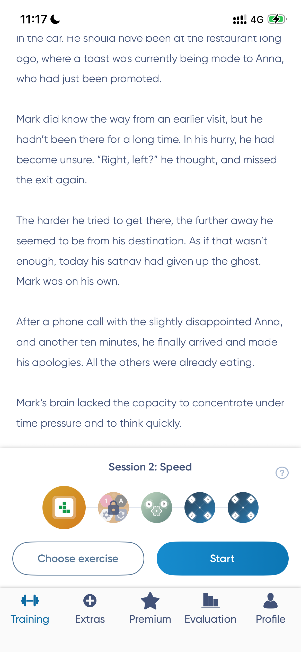

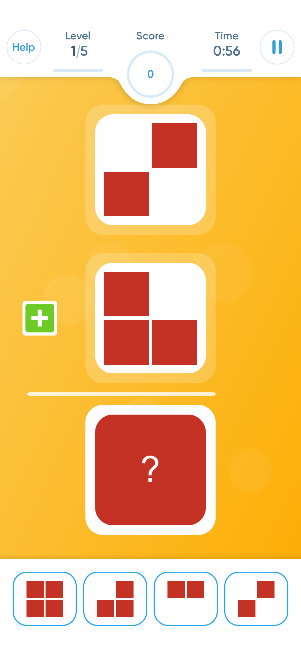

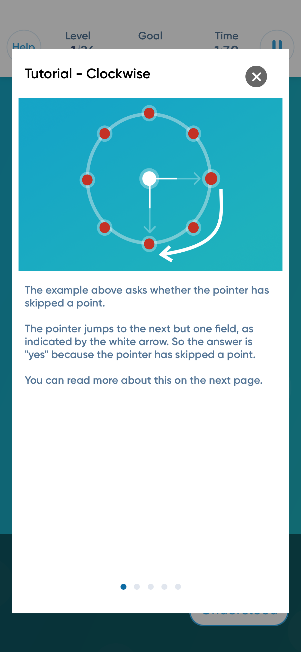

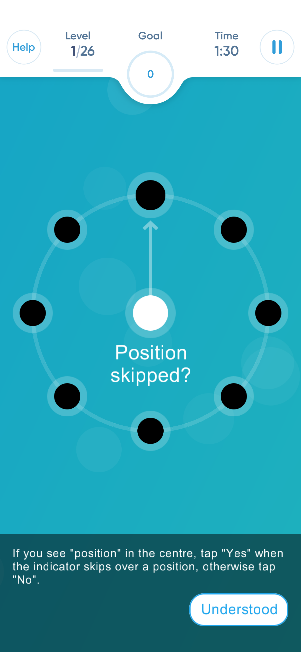


### Moodfit


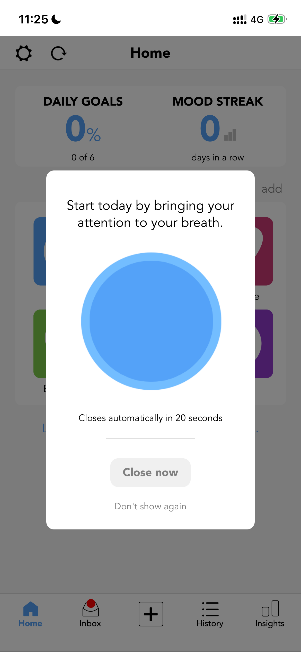

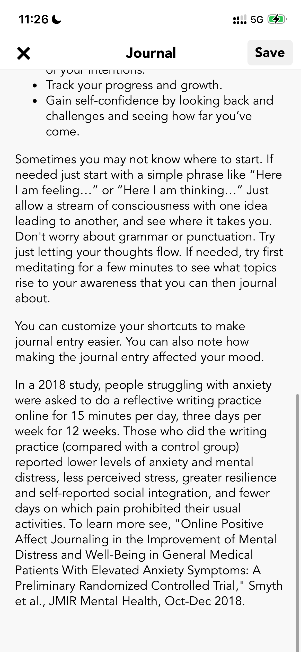

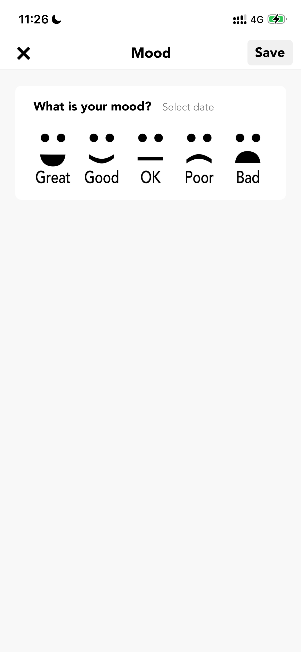

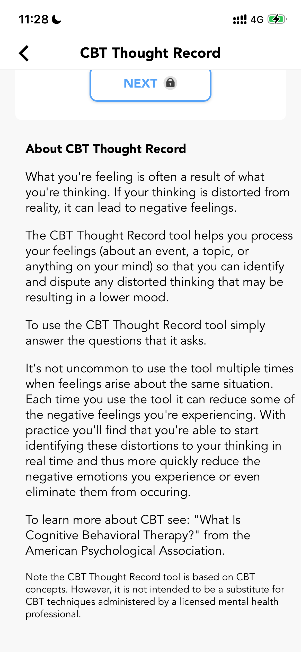


## R Package Citations

[[1]]

Wickham H, Averick M, Bryan J, Chang W, McGowan LD, François

R, Grolemund G, Hayes A, Henry L, Hester J, Kuhn M, Pedersen

TL, Miller E, Bache SM, Müller K, Ooms J, Robinson D, Seidel

DP, Spinu V, Takahashi K, Vaughan D, Wilke C, Woo K, Yutani H

(2019). “Welcome to the tidyverse.” _Journal of Open Source

Software_, *4*(43), 1686. doi:10.21105/joss.01686

<https://doi.org/10.21105/joss.01686>.

[[2]]

Wickham H (2016). _ggplot2: Elegant Graphics for Data

Analysis_. Springer-Verlag New York. ISBN 978-3-319-24277-4,

<https://ggplot2.tidyverse.org>.

[[3]]

Tingley D, Yamamoto T, Hirose K, Keele L, Imai K (2014).

“mediation: R Package for Causal Mediation Analysis.” _Journal

of Statistical Software_, *59*(5), 1-38.

<http://www.jstatsoft.org/v59/i05/>.

Imai K, Keele L, Yamamoto T (2010). “Identification,

Inference, and Sensitivity Analysis for Causal Mediation

Effects.” _Statistical Science_, *25*(1), 51-71.

<http://imai.princeton.edu/research/mediation.html>.

Imai K, Keele L, Tingley D (2010). “A General Approach to

Causal Mediation Analysis.” _Psychological Methods_, *15*(4),

309-334. <http://imai.princeton.edu/research/BaronKenny.html>.

Imai K, Keele L, Tingley D, Yamamoto T (2011). “Unpacking the

Black Box of Causality: Learning about Causal Mechanisms from

Experimental and Observational Studies.” _American Political

Science Review_, *105*(4), 765-789.

<http://imai.princeton.edu/research/mediationP.html>.

Imai K, Yamamoto T (2013). “Identification and Sensitivity

Analysis for Multiple Causal Mechanisms: Revisiting Evidence

from Framing Experiments.” _Political Analysis_, *21*(2),

141-171. <http://imai.princeton.edu/research/medsens.html>.

Imai K, Keele L, Tingley D, Yamamoto T (2010). “Causal

Mediation Analysis Using R.” In Vinod HD (ed.), _Advances in

Social Science Research Using R_. Springer-Verlag, New York.

[[4]]

van Buuren S, Groothuis-Oudshoorn K (2011). “mice:

Multivariate Imputation by Chained Equations in R.” _Journal

of Statistical Software_, *45*(3), 1-67.

doi:10.18637/jss.v045.i03

<https://doi.org/10.18637/jss.v045.i03>.

[[5]]

Bates D, Mächler M, Bolker B, Walker S (2015). “Fitting Linear

Mixed-Effects Models Using lme4.” _Journal of Statistical

Software_, *67*(1), 1-48. doi:10.18637/jss.v067.i01

<https://doi.org/10.18637/jss.v067.i01>.

[[6]]

Kuznetsova A, Brockhoff PB, Christensen RHB (2017). “lmerTest

Package: Tests in Linear Mixed Effects Models.” _Journal of

Statistical Software_, *82*(13), 1-26.

doi:10.18637/jss.v082.i13

<https://doi.org/10.18637/jss.v082.i13>.

[[7]]

Thériault R (2022). “rempsyc: Convenience Functions for

Psychology.” (R package version 0.1.3) [Computer software].,

<https://rempsyc.remi-theriault.com>.

[[8]]

Dayim A (2024). _consort: Create Consort Diagram_. R package

version 1.2.2, <https://CRAN.R-project.org/package=consort>.

[[9]]

R Core Team (2022). _R: A Language and Environment for

Statistical Computing_. R Foundation for Statistical

Computing, Vienna, Austria. <https://www.R-project.org/>.

[[10]]

Pedersen T (2024). _patchwork: The Composer of Plots_. R

package version 1.2.0,

<https://CRAN.R-project.org/package=patchwork>.

[[11]]

Iannone R, Roy O (2024). _DiagrammeR: Graph/Network

Visualization_. R package version 1.0.11,

<https://CRAN.R-project.org/package=DiagrammeR>.

[[12]]

Iannone R (2016). _DiagrammeRsvg: Export DiagrammeR Graphviz

Graphs as SVG_. R package version 0.1,

<https://CRAN.R-project.org/package=DiagrammeRsvg>.

[[13]]

Zeileis A, Hothorn T (2002). “Diagnostic Checking in

Regression Relationships.” _R News_, *2*(3), 7-10.

<https://CRAN.R-project.org/doc/Rnews/>.

[[14]]

Pinheiro J, Bates D, R Core Team (2023). _nlme: Linear and

Nonlinear Mixed Effects Models_. R package version 3.1-162,

<https://CRAN.R-project.org/package=nlme>.

Pinheiro JC, Bates DM (2000). _Mixed-Effects Models in S and

S-PLUS_. Springer, New York. doi:10.1007/b98882

<https://doi.org/10.1007/b98882>.

[[15]]

Tierney N, Cook D (2023). “Expanding Tidy Data Principles to

Facilitate Missing Data Exploration, Visualization and

Assessment of Imputations.” _Journal of Statistical Software_,

*105*(7), 1-31. doi:10.18637/jss.v105.i07

<https://doi.org/10.18637/jss.v105.i07>.

[[16]]

William Revelle (2025). _psych: Procedures for Psychological,

Psychometric, and Personality Research_. Northwestern

University, Evanston, Illinois. R package version 2.5.3,

<https://CRAN.R-project.org/package=psych>.

[[17]]

Champely S (2020). _pwr: Basic Functions for Power Analysis_.

R package version 1.3-0,

<https://CRAN.R-project.org/package=pwr>.

[[18]]

Mangiafico SS (2024). _rcompanion: Functions to Support

Extension Education Program Evaluation_. Rutgers Cooperative

Extension, New Brunswick, New Jersey. version 2.4.35,

<https://CRAN.R-project.org/package=rcompanion/>.

[[19]]

Navarro D (2015). _Learning statistics with R: A tutorial for

psychology students and other beginners. (Version 0.6)_.

University of New South Wales, Sydney, Australia. R package

version 0.5.1, <https://learningstatisticswithr.com>.

[[20]]

Robinson D, Hayes A, Couch S (2023). _broom: Convert

Statistical Objects into Tidy Tibbles_. R package version

1.0.5, <https://CRAN.R-project.org/package=broom>.

[[21]]

Lenth R (2023). _emmeans: Estimated Marginal Means, aka

Least-Squares Means_. R package version 1.8.7,

<https://CRAN.R-project.org/package=emmeans>.

[[22]]

Makowski D, Wiernik B, Patil I, Lüdecke D, Ben-Shachar M

(2022). “correlation: Methods for Correlation Analysis.”

Version 0.8.3,

<https://CRAN.R-project.org/package=correlation>.

Makowski D, Ben-Shachar M, Patil I, Lüdecke D (2020). “Methods

and Algorithms for Correlation Analysis in R.” _Journal of

Open Source Software_, *5*(51), 2306. doi:10.21105/joss.02306

<https://doi.org/10.21105/joss.02306>,

<https://joss.theoj.org/papers/10.21105/joss.02306>.

[[23]]

Kassambara A (2023). _rstatix: Pipe-Friendly Framework for

Basic Statistical Tests_. R package version 0.7.2,

<https://CRAN.R-project.org/package=rstatix>.

[[24]]

Kassambara A (2023). _ggpubr: 'ggplot2' Based Publication

Ready Plots_. R package version 0.6.0,

<https://CRAN.R-project.org/package=ggpubr>.

[[25]]

Wickham H (2023). _modelr: Modelling Functions that Work with

the Pipe_. R package version 0.1.11,

<https://CRAN.R-project.org/package=modelr>.

[[26]]

Harrell Jr F (2024). _Hmisc: Harrell Miscellaneous_. R package

version 5.1-3, <https://CRAN.R-project.org/package=Hmisc>.

[[27]]

Makowski D, Lüdecke D, Patil I, Thériault R, Ben-Shachar M,

Wiernik B (2023). “Automated Results Reporting as a Practical

Tool to Improve Reproducibility and Methodological Best

Practices Adoption.” _CRAN_.

<https://easystats.github.io/report/>.

[[28]]

Lüdecke D (2018). “ggeffects: Tidy Data Frames of Marginal

Effects from Regression Models.” _Journal of Open Source

Software_, *3*(26), 772. doi:10.21105/joss.00772

<https://doi.org/10.21105/joss.00772>.

[[29]]

Demin G (2023). _expss: Tables, Labels and Some Useful

Functions from Spreadsheets and 'SPSS' Statistics_. R package

version 0.11.6, <https://CRAN.R-project.org/package=expss>.

[[30]]

Long JA (2022). _jtools: Analysis and Presentation of Social

Scientific Data_. R package version 2.2.0,

<https://cran.r-project.org/package=jtools>.

## Supplementary Analyses

### FDR adjusted p values for sensitivity analyses

| Table S1. FDR adjusted p values for recovery and minimal clinically important change tests | | | | | | |
| --- | --- | --- | --- | --- | --- | --- |
|  |  | Executive function training | | Self-guided CBT | |  |
| Model |  | *exp(b)* [95%CI] | Adj. *P* | *exp(b)* [95% CI] | Adj. *P* |  |
| PHQ-9 Recovery | 4 weeks | **4.77 [1.59, 14.30]** | **.040** | **3.82 [1.25, 11.69]** | **.046** |  |
| PHQ-9 Recovery | 12 weeks | **5.33 [1.28, 22.19]** | **.046** | 2.00 [0.49, 8.24] | .449 |  |
| PHQ-9 MCID | 4 weeks | **3.54 [1.19, 10.50]** | .**046** | **3.66 [1.13, 10.88]** | **.046** |  |
| PHQ-9 MCID | 12 weeks | 1.78 [0.44, 7.18] | .479 | 0.83 [0.21, 3.35] | .797 |  |
| GAD-7 Recovery | 4 weeks | **3.75 [1.38, 10.17]** | **.036** | **1.96 [1.58, 12.14]** | **.036** |  |
| GAD-7 Recovery | 12 weeks | 3.18 [0.72, 12.94] | .169 | 3.50 [0.85, 14.41] | .166 |  |
| GAD-7 MCID | 4 weeks | 1.84 [0.69, 4.87] | .254 | **3.13 [1.07, 9.09]** | .096 |  |
| GAD-7 MCID | 12 weeks | 1.33 [0.38, 4.73] | .656 | 2.33 [0.62, 8.82] | .253 |  |
| PHQ-9 = Patient Health Questionnaire; GAD-7 = Generalised Anxiety Disorder Assessment. Waitlist control was set as the reference group. | | | | | |  |

### Results of primary outcome regression analyses with post-training or follow-up score as the outcome variable, controlling for baseline score

| Table S2. Coefficients of regression analyses controlling for baseline depression or anxiety. | | | | | | |
| --- | --- | --- | --- | --- | --- | --- |
|  |  | Neuronation | | Moodfit | |  |
| Outcome |  | *B* [95%CI] | *p* | *B* [95% CI] | *p* |  |
| PHQ-9 | 4 weeks | -1.07 [-2.82, 0.67] | .226 | -1.65 [-3.39, 0.10] | .064 |  |
| PHQ-9 | 12 weeks | -2.26 [-4.58, 0.06] | .056 | -1.31 [-3.36, 0.99] | .262 |  |
| GAD-7 | 4 weeks | -1.13 [-2.56, 0.30] | .121 | -1.23 [-2.66, 0.20] | .092 |  |
| GAD-7 | 12 weeks | **-2.54 [-4.54, -0.55]** | **.013** | -1.80 [-3.78, 0.17] | .073 |  |
| UWES-9 | 4 weeks | 2.97 [-0.20, 6.13] | .066 | **3.97 [0.83, 7.11]** | **.013** |  |
| UWES-9 | 12 weeks | 3.47 [-1.34, 8.27] | .155 | 4.52 [-0.22, 9.26] | .062 |  |
| Note: Bold denotes significance p < .05 | | | | | |  |

### Results of regression models of proportion of letters against depressive symptoms at each set size

| Table S3. OSPAN task performance predicting depressive symptoms at each set size. | | | | |
| --- | --- | --- | --- | --- |
| Set Size | *b* | SE | *p* | 95% CI |
| 3 | 0.08 | 0.78 | .921 | [-1.46, 1.61] |
| 4 | 0.93 | 0.71 | .196 | [-0.48, 2.33] |
| 5 | -0.07 | 0.63 | .908 | [-1.30, 1.16] |
| 6 | -0.04 | 0.60 | .949 | [-1.21, 1.14] |
| 7 | -0.73 | 0.43 | .092 | [-1.58, 0.12] |

### Results of regression models of proportion of letters against anxious symptoms at each set size

| Table S4. OSPAN task performance predicting anxious symptoms at each set size. | | | | |
| --- | --- | --- | --- | --- |
| Set Size | *b* | SE | *p* | 95% CI |
| 3 | -0.35 | 0.70 | .617 | [-1.72, 1.02] |
| 4 | 0.78 | 0.64 | .222 | [-0.47, 2.04] |
| 5 | 0.20 | 0.56 | .725 | [-0.90, 1.30] |
| 6 | 0.36 | 0.54 | .506 | [-0.70, 1.41] |
| 7 | -0.33 | 0.39 | .401 | [-1.09, 0.44] |

### Coefficients for logistic regressions of intervention effects on applied workplace outcomes

| Table S5. Logistic regression coefficients for intervention effects on workplace stress, presenteeism, absenteeism and leaveism. | | | | |
| --- | --- | --- | --- | --- |
|  | Neuronation | | Moodfit | |
| Outcome | *exp(b)* [95%CI] | *p* | *exp(b)* [95% CI] | *p* |
| Stressful events | 0.48 [0.19, 1.20] | .117 | 0.71 [0.29, 1.74] | .455 |
| Presenteeism | 0.59 [0.15, 2.29] | .443 | 2.17 [0.69, 6.86] | .185 |
| Absenteeism | 1.38 [0.32, 5.88] | .667 | 2.57 [0.56, 11.72] | .224 |
| Leaveism | 1.11 [0.36, 3.44] | .863 | 1.80 [0.59, 5.47] | .299 |
| Note. Presenteeism, Absenteeism and Leaveism (working on leave) coded so higher odds ratios indicate less chance of event occurring. | | | | |
